# Ceftazidime/avibactam combined with colistin: a novel attempt to treat carbapenem-resistant Gram-negative bacilli infection

**DOI:** 10.1186/s12879-023-08715-w

**Published:** 2023-10-20

**Authors:** Zihao Zheng, Ziqiang Shao, Lihai Lu, Siyu Tang, Kai Shi, Fangxiao Gong, Jingquan Liu

**Affiliations:** 1 Fourth School of Clinical Medicine, Zhejiang Chinese Medical University, Hangzhou, 310053 Zhejiang Province China; 2 Emergency and Critical Care Center, Intensive Care Unit, Zhejiang Provincial People’s Hospital, Affiliated People’s Hospital, Hangzhou Medical College, No. 158 Shangtang Road, Gongshu District, Hangzhou, 310014 Zhejiang Province China; 3 The Second School of Clinical Medicine, Zhejiang Chinese Medical University, Hangzhou, 310053 Zhejiang Province China; 4 Department of Respiratory Medicine, The Affiliated Hospital of Hangzhou Normal University, Hangzhou, 310015 Zhejiang Province China

**Keywords:** Ceftazidime/avibactam, Colistin, Combination therapy, Carbapenem-resistant Gram-negative bacilli

## Abstract

**Background:**

The rapid global emergence and spread of carbapenem-resistant Gram-negative bacilli (CR-GNB) is recognized as a major public health concern, and there are currently few effective treatments for CR-GNB infection. The aim of this study was to investigate the clinical characteristics and outcomes of patients with CR-GNB infections treated with ceftazidime/avibactam (CAZ/AVI) combined with colistin from October 2019 to February 2023 in China.

**Methods:**

A total of 31 patients with CR-GNB infections were retrospectively identified using the electronic medical record system of Zhejiang Provincial People's Hospital.

**Results:**

Thirty-one patients were treated with CAZ/AVI combined with colistin. Respiratory tract infections (87%) were most common. The common drug-resistant bacteria encompass Klebsiella pneumonia (54.8%), Acinetobacter baumannii (29.0%), and Pseudomonas aeruginosa (16.1%). The 30-day mortality rate was 29.0%, and the 7-day microbial clearance rate was 64.5%. The inflammatory marker CRP changes, but not PCT and WBC, were statistically significant on days 7 and 14 after combination therapy. There were seven patients developing acute renal injury (AKI) after combination therapy and treating with continuous renal replacement therapy (CRRT). Two patients developed diarrhea.

**Conclusion:**

The combination of CAZ/AVI and colistin has potential efficacy in patients with CR-GNB infection, but more studies are needed to determine whether it can reduce 30-day mortality rates and increase 7-day microbial clearance. At the same time, the adverse reactions of combination therapy should not be ignored.

## Background

The rapid global emergence and spread of multidrug-resistant (MDR) and extensively drug-resistant (XDR) Gram-negative bacteria (GNB), particularly carbapenem-resistant Gram-negative bacilli (CR-GNB), is recognized as a major public health concern [1–4]. It is predicted that the number of bacterial infections is expected to reach 10 million by about 2050 [5], indicating that bacterial resistance has become a significant problem that cannot be ignored. In 2017, WHO published a list of bacteria for which new antimicrobials are urgently needed. Carbapenem-resistant Gram-negative bacilli, such as carbapenem-resistant Acinetobacter baumannii, carbapenem-resistant Pseudomonas aeruginosa, carbapenem-resistant Enterobacteriaceae, and broad-spectrum β-lactamase-producing Enterobacteriaceae, was identified as crucial pathogens [6]. These pathogenic bacteria are fatal factors causing septic shock [7], severe pneumonia [8], and acute kidney injury [9].

The production of carbapenemases is the primary resistance mechanism of carbapenem-resistant CR-GNB. According to the Ambler classification system, carbapenemases can be divided into classes A, B, and D β-lactamases [10]. Class A carbapenemases use serine residues to hydrolyze β-lactamases, including the blaKPC, blaNMC /blaIMI, and blaSME genes, of which blaKPC is the most common carbapenemase in this class and is mainly found in Klebsiella pneumoniae [10, 11]. Class B metalloid beta-lactamases (MBLs) are zinc-dependent and include the blaVIM, blaIMP, and blaNDM genes. Impasse was the first enzyme identified in this class and now accounts for 15% of CRE found in Japan, Australia, and parts of Southeast Asia, according to Matsumura, Y's report on IMP-producing Enterobacteriaceae worldwide [12]. Recently, the rapid spread of New Delhi metallo-β-lactamase (NDM) and the limitation of treatment have attracted wide attention [13]. Class D carbapenemases include members of the OXA-encoding gene and are mainly found in Acinetobacter. The common OXA-encoding genes are OXA-48-like enzymes, including their related variants, such as OXA-181, OXA-162, and OXA-232, mainly found in Europe and the Middle East [14, 15]. The production of the carbapenemases mentioned above is the leading cause of resistance to common antibiotics such as meropenem and imipenem in clinics.

Unfortunately, there are currently few effective treatments for CR-GNB infection. The generally accepted one is colistin, whose antibacterial mechanism mainly involves destruction of the outer membrane, resulting in leakage of bacterial cytoplasmic contents or the neutralization of GNB endotoxins corresponding to the lipid A part of lipopolysaccharides [16, 17]. However, colistin resistance has been increasing recently, and even studies have found that colistin resistance is associated with increased mortality. The recent literature published by Tompkins, K suggested that the efficacy of polymyxin antibiotics against bacteria producing class A, B, and D carbapenemases is limited, and nephrotoxicity is evident [10], which also increased the concern of doctors about colistin treatment of CR-GNB to a certain extent. Recently, ceftazidime-avibactam (CZA/AVI) has emerged as the treatment of choice for resistant bacteria, especially for CR-GNB infections, and it belongs to a relatively new combination of a third-generation cephalosporin and a novel β-lactamase inhibitor [18]. Avibactam can reversibly bind to β-lactamase (OXA-48), effectively inactivating β-lactamase and preventing the hydrolysis of β-lactam compounds. However, traditional β-lactamase inhibitors and other non-β-lactam inhibitors do not inhibit OXA-48. In addition, AVI also inhibits extended spectrum β-lactamases (ESBLs) and class C cephalosporins, providing a potential treatment option for infections caused by multidrug-resistant Gram-negative pathogens [19]. CAZ/AVI was approved in Europe in 2016, followed by Russia (2017) and Latin American countries (2018 in Argentina and Brazil, 2019 in Columbia) for the treatment of adults with complicated urinary tract infection, complicated intra-abdominal infection, and hospital-acquired pneumonia/ventilator-associated pneumonia, with an overall success rate of over 70% for CR-GNB infection [20], so its clinical application is gradually increasing. However, several in vitro and in vivo studies have demonstrated increasing resistance to CAZ/AVI along with their increased use. In fact, AVI has no activity against class B carbapenemase-producing bacteria [10], and thus, CAZ/AVI is not sensitive to some drug-resistant bacteria.

Obviously, treating CR-GNB infection should not be limited to monotherapy with CAZ/AVI or colistin alone. Previous studies have shown that the combination of antibiotics can effectively treat infections caused by multi-drug-resistant bacteria [21]. In addition, according to the above content, we have learned that the mechanism of action of the two antibiotics is different, and the combination of the two antibiotics may has a broader bactericidal effect. However, the combination of the two drugs is rare in clinical practice, and the accuracy of its efficacy needs to be further explored. Therefore, we retrospectively collected and analyzed the data related to the combination of CAZ/AVI and colistin in the treatment of CR-GNB infection in order to explore the efficacy of the combination of the two. At the same time, we evaluated their safety to weigh the benefits and harms of combination therapy clinically.

## Materials and methods

### Study design and population

**Fig. 1:**
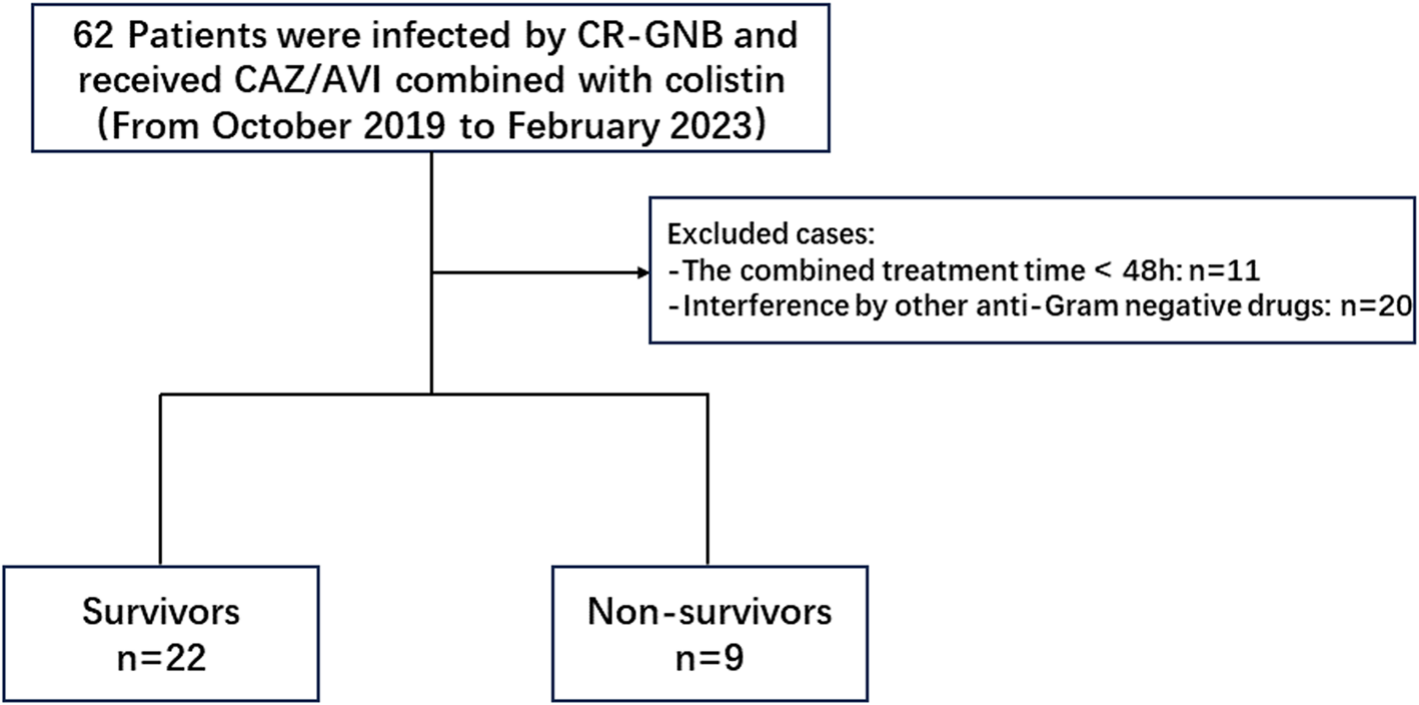
Flow chart of the study. CR-GNB, carbapenem-resistant Gram-negative bacilli; CAZ/AVI, Ceftazidime-avibactam

tjdgld retrospective study conducted from October 2019 to February 2023 in the general intensive care unit (50 beds) of Zhejiang Provincial People's Hospital, a Class 3 teaching hospital with over 3000 beds in Hangzhou, Zhejiang Province, China. The use of CAZ/AVI and colistin during this period followed guideline recommendations [22, 23]. The dosage and mode of the combination were as follows: CAZ/AVI (2.5 g q8h) and colistin (750,000 IU q12h) by intravenous titration. Patients aged ≥ 18 years who were infected by CR-GNB and received CAZ/AVI combined with colistin for ≥ 48 h were included (Fig. 1). This study was approved by the Institutional Review Board and the Ethics Committee of the of Zhejiang Provincial People's Hospital, which complies with the Declaration of Helsinki (ethics approval number: QT2023178, Date of approval: 29/05/2023). And individual consent for this retrospective analysis was waived.

### Identification of Micro-organisms

Identification of micro-organisms was performed by matrix-assisted laser desorption/ionization time-of-flight mass spectrometry, and the type of carbapenemase (e.g., SBL or MBL) was identified by the modified carbapenem inactivation method. Minimum inhibitory concentrations (MICs) were based on Clinical and Laboratory Standards Institute (CLSI) guidelines [24].

### Date collection

Patient data were collected from the electronic medical record system of the hospital. Baseline data included: demographic characteristics, underlying diseases and comorbidities, SOFA and Apache II scores, site and type of infection, days of colistin use before combined treatment, use of vasoactive agents and modes of respiratory support. For enrolled patients, the time from the beginning of infection to the start of treatment, as well as the duration of treatment, microbial culture results, and changes in inflammatory markers during the treatment period were recorded. In addition, outcome variables included total lengths of hospital and ICU stay, as well as treatment outcome (30-day mortality rate, 7-day microbial clearance rate, changes in inflammatory markers). Safety evaluation mainly refers to nephrotoxicity but includes diarrhea, nervous system toxicity, and anaphylaxis.

### Definition

Carbapenem resistance was defined as a MIC of imipenem or meropenem ≥ 4 mg/L. The types of infections were defined according to the standardized definitions of the Centers for Disease Control and Prevention’s National Healthcare Safety Network [25]. The enrollment time was defined as 24 h before combination therapy. Length of ICU stay was defined as the period from enrollment time to discharge or death. Mortality within 30 days was calculated from enrollment time. Microbial clearance was defined as absence of the initially isolated pathogen from microbial cultivation by the 7th day.

## Data analysis

Data analysis was performed using IBM SPSS Statistics v.25.0 (IBM Corp., Armonk, NY, USA). Continuous variables were compared using Student’s t-test and categorical variables using Fisher’s exact test. We established cut-off values for days of combination therapy and nephrotoxicity in our population based on the characteristics of patients as previously described. Graph plotting was performed using GraphPad Prism v.8.03 (GraphPad Software, Inc., La Jolla, CA, USA). Statistical significance was defined as a two-tailed *P*-value of ＜ 0.05.

## Result

### Clinical and microbiological characteristics

A total of 31 consecutive patients treated with CAZ/AVI combined with colistin were enrolled in this study. The mean ± standard deviation patient age and BMI were 71.23 ± 12.67 years and 22.78 ± 3.92 kg/m^2^, respectively. Among all patients, 25 (80.6%) were male, and 17 and 11 had hypertension and diabetes, respectively. Twenty-eight patients required ventilator-assisted ventilation, and three required high-flow nasal cannula oxygen therapy. The Apache II and SOFA scores at enrollment were 22.26 ± 7.29 and 10.32 ± 3.67, respectively.

The types of infections included respiratory tract infections (27/31, 87%), urinary tract infections, and bloodstream infections (both 2/31, 6.5%). Pathogens included carbapenem-resistant K. pneumoniae (17/31, 54.8%), carbapenem-resistant A. baumannii (9/31, 29.0%), and carbapenem-resistant P. aeruginosa (5/31, 16.1%). The types of carbapenemases included KPC (15/31, 48.4%), IMP (4/31, 12.9%), NDM (3/31, 9.7%), OXA-23 (6/31, 19.3%), and OXA-48 (3/31, 9.7%). In addition, with subsequent disease progression, 24 patients developed sepsis and 22 developed septic shock. Table 1 describes the statistical data of the 31 patients.

**Table 1:** Baseline characteristics of patients with infections caused by carbapenem-resistant Gram-negative bacilli

Characteristics	Total (*n* = 31)	Survivors (*n* = 22)	Non-Survivors (*n* = 9)	*P*
Demographic variables				
Age (mean ± S.D.)	71.23 ± 12.67	67.55 ± 11.84	80.22 ± 10.26	0.009
Male [n (%)]	25(80.6)	17(77.3)	8(88.9)	0.642
BMI (mean ± S.D.)	22.78 ± 3.92	22.92 ± 3.97	22.42 ± 4.00	0.753
Smoke [n (%)]	7(22.5)	6(27.3)	1(11.1)	0.639
Drink [n (%)]	4(12.9)	3(13.6)	1(11.1)	1.000
Apache II score (mean ± S.D.)	22.26 ± 7.29	22.23 ± 7.45	22.33 ± 7.31	0.971
SOFA score (mean ± S.D.)	10.32 ± 3.67	10.45 ± 3.85	10.00 ± 3.39	0.760
Breath support [n (%)]				0.537
Mechanical ventilation	28(90.3)	20(90.9)	8(88.9)	
High frequency jet ventilation	3(9.7)	2(9.1)	1(11.1)	
Underlying diseases [n (%)]				
Hypertension	17(54.8)	15(68.2)	2(22.2)	0.044
Diabetes	11(35.5)	9(40.9)	2(22.2)	0.429
Type of infection [n (%)]				0.063
Respiratory tract	27(87.1)	21(95.5)	6(66.7)	
Urinary tract	2(6.5)	1(4.5)	1(11.1)	
Bloodstream	2(6.5)	0	2(22.2)	
Pathogen [n (%)]				
CRKP	17(54.8)	14(63.6)	3(33.3)	0.233
CRPA	5(16.1)	4(18.2)	1(11.1)	1.000
CRAB	9(29.0)	4(18.2)	5(55.6)	0.077
Carbapenemases [n (%)]				
KPC	15(48.4)	13(59.1)	2(22.2)	0.113
IMP	4(12.9)	3(13.6)	1(11.1)	1.000
NDM	3(9.7)	2(9.1)	1(11.1)	1.000
OXA-23	6(19.3)	2(9.1)	4(44.4)	0.043
OXA-48	3(9.7)	2(9.1)	1(11.1)	1.000
Clinical presentation [n (%)]				
Sepsis	24(77.4)	17(77.3)	7(77.8)	1.000
Sepsis shock	22(71.0)	16(72.7)	6(66.7)	1.000
Comorbidities [n (%)]				
Severe pneumonia	10(32.3)	8(36.4)	2(22.2)	0.677
Respiratory failure	12(38.7)	9(40.9)	3(33.3)	1.000
Renal insufficiency	20(64.5)	14(63.6)	6(66.7)	1.000
Gastrointestinal bleeding	8(25.8)	5(22.7)	3(33.3)	0.660
Days of therapy (mean ± S.D.)	8.16 ± 3.39	8.50 ± 4.74	7.33 ± 3.46	0.511
Days of colistin treatment before combination therapy (median [25%, 75%])	6.00[1.00, 12.00]	6.00[0.75, 12.00]	4.00[1.5, 11.5]	0.643
Vasoactive agent [n (%)]				
Norepinephrine	21(67.7)	15(68.2)	6(66.7)	1.000
Aramine	8(25.8)	6(27.3)	2(22.2)	1.000
Hypophysin	8(25.8)	4(18.2)	4(44.4)	0.185
Outcome				
Length of hospital stay (mean ± S.D.)	39.06 ± 19.43	40.45 ± 18.96	35.67 ± 21.33	0.568
Length of ICU stay (mean ± S.D.)	17.06 ± 13.48	19.68 ± 14.86	10.67 ± 6.04	0.023
Microbial clearance within seven days [n (%)]	20(64.5)	16(72.7)	4(44.4)	0.217
Safety evaluation				
AKI [n (%)]	7(22.6)	6(27.3)	1(11.1)	0.639
Diarrhea [n (%)]	2(6.5)	2(6.5)	0	1.000

*S.D.* standard deviation, *BMI* Body mass index, *Apache* Acute Physiology and Chronic Health Evaluation, *SOFA* Sequential Organ Failure Assessment, *ICU* intensive care unit. Non-survivors, patients who died within 30 days; *CRKP* carbapenem-resistant Klebsiella pneumoniae, *CRAP* carbapenem-resistant Pseudomonas aeruginosa, *CRAB* carbapenem-resistant Acinetobacter baumanni, *AKI* acute kidney injury

### Antibiotic and clinical effects

Twenty-four patients received colistin for a median of 6 days before enrollment. The average colistin and CAZ/AVI combination therapy duration was eight days after enrollment. During the combination therapy, caspofungin and voriconazole were added in 10 and 4 patients, respectively, and linezolid, teicoplanin, and vancomycin were added in 3, 2, and 1 patient, respectively. The use of antibiotics in the survival group is described in Table 2.

**Table 2:** Clinical characteristics of survivors (*n* = 22)

Patient	Gender	Age (years), BMI (kg/m^2^)	Cause of Hospitalization	Use of vasoactive agents	Use of other antibiotics during combination therapy	Length of treatment days with combined therapy (day)	Days of colistin treatment before combination therapy (day)	Type of infection	Pathogen, carbapenemases	Apache II/SOFA score	Total length of stay (days)	length of stay in ICU (days)	Microbial cure within seven days	Adverse events
1	M	68, 18.4	Gastric malignant tumor	NE 6 mg q12h for 11 days	No	13	8	Respiratory tract	CRKP, IMP	15/3	54	14	Y	N
2	M	79, 23.9	Severe pneumonia	NE 4 mg q12h for 3 days	CAS 50 mg qd for 5 days	12	17	Respiratory tract	CRKP, KPC	23/9	52	31	N	AKI
3	M	61, 20.8	Hemorrhagic shock	ARA 100 mg qd for 10 days	No	3	6	Respiratory tract	CRKP, KPC	16/16	16	4	Y	N
4	M	61, 32.3	Respiratory failure	NE 4 mg q12h for 3 days	No	3	6	Respiratory tract	CRKP, IMP	20/9	19	4	N	N
5	M	77, 21.5	Spinal cord injury	NE 20 mg q12h for 13 days	No	3	0	Urinary tract	CRAB, OXA-23	20/7	55	34	Y	N
7	F	75, 26.0	Ileus	No	CAS 50 mg qd for 12 days	16	3	Respiratory tract	CRAB, OXA-23	21/9	45	32	Y	N
8	M	62, 26.8	Sepsis	NE 6 mg q12h for 10 days	CAS 50 mg qd for 5 days	11	19	Respiratory tract	CRPA, KPC	13/12	36	12	Y	N
10	M	56, 24.2	Decompensated liver cirrhosis	NE 10 mg q12h and HYP 18iu q12h for 7 days	CAS 50 mg qd for 7 days	7	6	Respiratory tract	CRKP, KPC	16/9	32	8	N	N
11	M	71,24.2	Cardiogenic shock	ARA 100 mg qd for 6 days	No	7	1	Respiratory tract	CRKP, KPC	23/12	27	12	Y	AKI
13	F	69, 23.8	Severe pneumonia	NE 20 mg q8h and HYP 18iu q8h for 5 days	CAS 50 mg qd for 3 days	3	5	Respiratory tract	CRPA, KPC	42/18	8	4	N	N
16	M	62, 29.4	Severe pneumonia	NE 20 mg q12h for 8 days	CAS 50 mg qd for 10 days	13	0	Respiratory tract	CRKP, KPC	25/10	52	40	Y	AKI and diarrhea
17	M	63, 25.0	Severe pneumonia	NE 6 mg q12h for 16 days	LIN 0.6 g q12h for 8 days	9	12	Respiratory tract	CRKP, IMP	20/10	35	22	Y	N
18	M	94, 18.0	Severe pneumonia	No	No	7	17	Respiratory tract	CRPA, KPC	30/9	70	54	Y	N
19	M	43, 21.2	Pulmonary infection	NE 6 mg q12h for 5 days	No	15	0	Respiratory tract	CRPA, KPC	18/7	86	44	Y	N
22	M	73, 26.4	Severe pneumonia	ARA 50 mg qd for 13 days and NE 20 mg q8h for 4 days	No	16	12	Respiratory tract	CRKP, KPC	14/8	32	17	Y	AKI
23	M	71, 22.5	Aspiration pneumonia	NE 10 mg q8h for 9 days	TEI 0.4 g q8h for 5 days	16	8	Respiratory tract	CRKP, KPC	38/14	49	18	Y	N
25	M	49, 19.6	Sepsis	No	No	5	5	Respiratory tract	CRAB, OXA-48	23/11	29	5	Y	AKI
26	F	84, 17.8	Sepsis	No	CAS 50 mg qd for 5 days	9	7	Respiratory tract	CRKP, NDM	26/7	56	37	N	AKI
27	M	64, 15.6	Renal insufficiency	No	CAS 50 mg qd for 16 days	6	10	Respiratory tract	CRKP, NDM	17/5	42	12	Y	N
28	M	76, 24.0	Sepsis	NE 6 mg q12h for 8 days	No	7	0	Respiratory tract	CRAB, OXA-48	31/16	27	10	Y	N
30	F	52, 20.0	Acute pancreatitis	NE 10 mg q8h for 7 days	VAN 1 g q12h for 4 days	3	0	Respiratory tract	CRKP, KPC	19/13	15	4	N	N
31	F	76, 22.8	Diabetic ketoacidosis	NE 4 mg q8h for 26 days	VOR 0.2 g q12h for 6 days	4	16	Respiratory tract	CRPA, KPC	19/16	53	15	Y	N

*M* Male, *F* Female, *Apache* Acute Physiology and Chronic Health Evaluation, *SOFA* Sequential Organ Failure Assessment, *ICU* intensive care unit, *NE* norepinephrine, *ARA* aramine, *HYP* hypophysin, *CRKP* carbapenem-resistant Klebsiella pneumoniae, *CRPA* carbapenem-resistant Pseudomonas aeruginosa, *CRAB* carbapenem-resistant Acinetobacter baumannii, *VAN* vancomycin, *LIN* linezolid, *CAS* caspofungin, *VOR* voriconazole, *TEI* teicoplanin, *AKI* acute renal injury, *Y* yes, *N* no

The 30-day mortality rate was 29.0%, and the average lengths of hospital and ICU stay were 39.0 and 17.0 days, respectively. The average lengths of ICU stay among the survivors and non-survivors were 19.7 and 10.7 days, respectively (P = 0.023). In addition, the pathogen was cleared within 7 days in 20 patients (20/31, 64.5%) (Table 1).

**Fig. 2:**
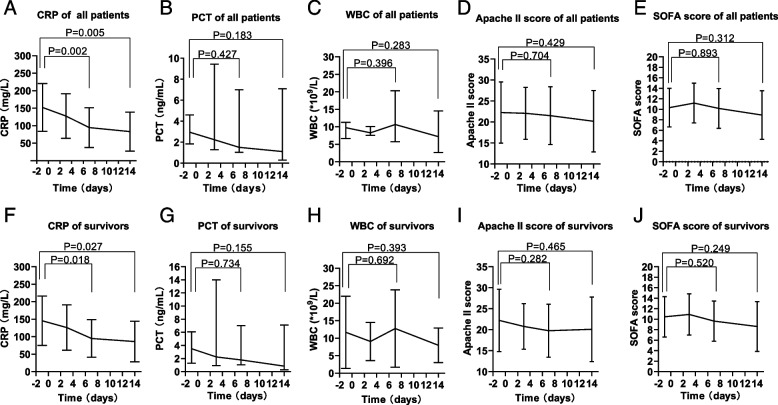
Dynamic changes of inflammatory markers and ICU-associated scores in CR-GNB patients. Changes in inflammatory markers and ICU-associated scores, including 1 day before and 3, 7, and 14 days after start of combination therapy. (**A**-**E**) CRP, PCT, WBC, Apache II and SOFA score of all patients (*n* = 31); (**F**-**J**) CRP, PCT, WBC, Apache II and SOFA score of survivors (*n* = 22)

Figure 2 shows the changes in inflammatory markers and ICU-associated scores among all patients and among survivors, including c-reactive protein (CRP), procalcitonin (PCT), white blood cell (WBC) levels, Apache II score and SOFA score 24 h before and 3, 7, and 14 days after the start of combination therapy. The mean ± standard deviation CRP level in all patients was 152.23 ± 68.42 mg/L within 24 h before combination therapy, 94.56 ± 56.86 mg/L (P = 0.002) on day 7 of combination therapy, and 83.20 ± 55.87 mg/L (P = 0.005) on day 14. Among survivors, the mean ± standard deviation CRP level was 145.61 ± 70.42 mg/L within 24 h before combination therapy, 94.82 ± 53.76 mg/L (P = 0.018) on day 7 of combination therapy, and 86.16 ± 57.98 mg/L (P = 0.027) on day 14. However, there were no significant changes in PCT or WBC levels among either all patients or survivors. For the change of ICU-associated scores, Apache II and SOFA scores showed a downward trend 24 h before treatment compared with seven days and fourteen days after combination treatment. However, there was no statistical difference.

**Fig. 3:**
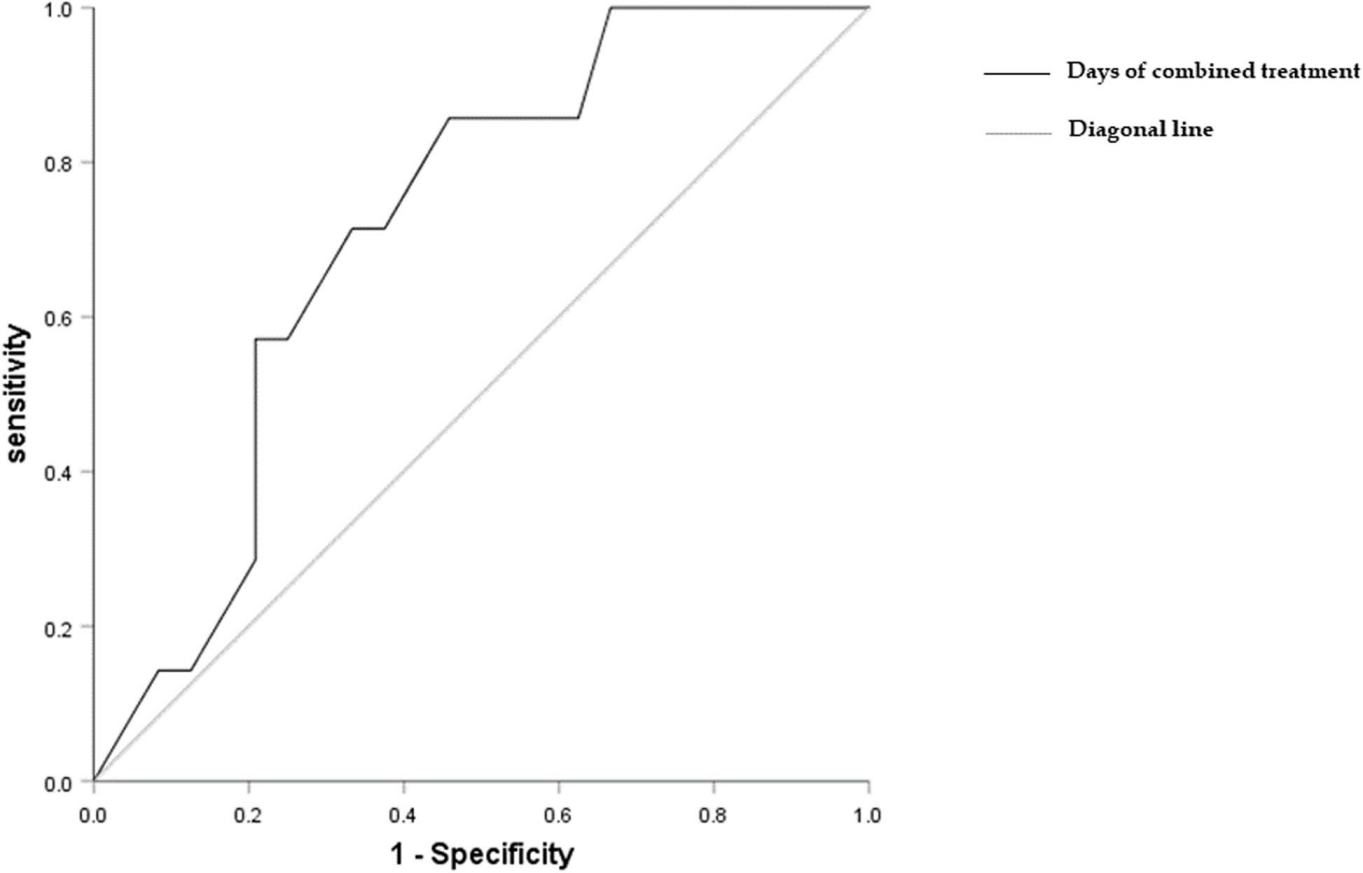
Receiver operating characteristic curve of the days of combined treatment in predicting acute kidney injury. The receiver operating curve (ROC) indicated that a cut-off value of the days of combined treatment was 8.5 days with 71.4% sensitivity and 66.7% specificity for predicting AKI in treating CR-GNB infection. AKI, acute kidney injury, ROC, receiver operating curve

For the safety evaluation results, AKI occurred in seven patients after combined therapy, and they were treated with continuous renal replacement therapy (CRRT). The receiver operating characteristic curve (ROC) was used to analyze the correlation between the days of combined treatment and AKI. The cut-off value was 8.5 days, the area under the curve (AUC) was 0.717, and the sensitivity, specificity, positive predictive value, and negative predictive value for nephrotoxicity were 71.4%, 66.7%, 38.5%, and 88.9%, respectively (Fig. 3). In addition, two patients had diarrhea (culture negative for Clostridium difficile), and no patient had seizures or headaches.

## Discussion

This was a single-center, real-world retrospective study of clinical treatment of CR-GNB infection with CAZ/AVI combined with colistin. According to our study, the combination therapy of 31 patients is beneficial to a certain extent, but we should also pay attention to the related adverse reactions. We present the actual outcomes such as 30-day mortality, 7-day bacterial clearance, change of inflammatory markers, and length of ICU stay from our study.

In the case of antibiotic resistance, combination therapy is a good option. Antibiotic combinations have the potential to improve outcomes by expanding the spectrum of antimicrobial activity, reducing the risk of resistance, and producing more substantial antimicrobial effects through synergy [26]. A meta-analysis of the in vitro efficacy of antibiotic combinations for CR-GNB by Scudeller, L et al. [27] concluded that colistin combined with fosfomycin and polymyxin combined with rifampin had increased bactericidal activity and decreased regrowth rate and that the combination of drugs had a synergistic effect. Similarly, Liu, X et al. [28] demonstrated a synergistic effect of colistin combined with meropenem against carbapenem-resistant Acinetobacter baumannii (CRAB) in vitro. However, since there may be differences in the pharmacokinetic effects of these drugs in the host, it is inaccurate to infer the clinical efficacy. More clinical studies are needed to explore the effectiveness of these combinations. The research conducted by Hao, M et al. [29] demonstrated that the utilization of colistin combination therapy for CR-GNB infection yielded superior outcomes in terms of bacterial clearance (74.1%), clinical response (94.4%), and 28-day mortality (5.6%) when compared to the use of colistin monotherapy. In 2021, Katip, W et al. [30] investigated the efficacy of colistin combined with vancomycin for treating CRAB. Through propensity matching analysis, the researchers determined that the combined treatment resulted in a 30-day mortality rate of 47.83%, a clinical effect of 58.26%, and a microbial clearance rate of 66.09%. In the subsequent year, the identical research team conducted a comparative study examining the efficacy of loading dose colistin in combination with meropenem versus loading dose colistin in combination with imipenem for treating CRAB infection [31]. The findings of this study revealed favorable clinical response rates (54.66% vs. 44.12%) and microbial clearance rates (62.38% vs. 54.41%). Additionally, the observed 30-day mortality rates were recorded (53.7% vs. 48.53%). In our study, the combination of CAZ/AVI and colistin resulted in an overall 7-day microbial clearance rate of 69% and a 30-day mortality rate of 29%. Compared with the previous findings of Hao, M and Katip, W et al., our efficacy was differential and slightly better than Wasan Katip et al. 's colistin combination regimen regarding microbial clearance and 30-day mortality. There may be the following reasons. First, 77.4% of the patients had been treated with colistin for some time before the combination, and longer colistin therapy may be superior to shorter colistin therapy [32]. Second, CRKP accounted for more than half (54.8%) of the CR-GNB types we included, and CAZ/AVI combined with colistin may be more effective in treating CRKP infection. Third, for patients with multiple bacterial infections during the combined treatment, we selected anti-Gram-positive bacteria and antifungal drugs with less impact on Gram-negative bacteria for treatment. There may be a synergistic effect between antibiotics, which affects the survival rate of patients to a certain extent. Therefore, the mortality and clearance rates in our study are not superior.

CAZ/AVI is mainly used to treat multi-drug resistant Gram-negative bacterial infections. Due to the late introduction of CAZ/AVI, few reports on CAZ/AVI related antibiotic combinations are still available. At present, most studies on CAZ/AVI combined with colistin for CR-GNB infection are in vitro antibiotic activity studies. For example, Mataraci et al. suggested that CAZ/AVI combined with colistin was effective against OXA-48-producing Enterobacterales in vitro [33]. A recent in vitro time–kill experiment by Wang et al. also concluded that combination therapy may be more beneficial than monotherapy in the treatment of carbapenemase-producing K. pneumonia [34]. For other drug-resistant strains, such as CRAB and CRPA, the in vitro activity of isolated strains still needs to be further studied to verify the hypothesis of clinical efficacy. In a recent multicenter clinical study of CAZ/AVI for CRKP infection, Tumbarello, M et al. [35] concluded that there was no statistically significant difference in 30-day mortality between CAZ/AVI monotherapy and combination therapy (26.1% vs. 25.0%, P = 0.79). This result is similar to a previous meta-analysis published by Fiore, M [36]. However, this does not deny the efficacy of CAZ/AVI combined with colistin in the clinic. After all, CAZ/AVI combined with colistin only accounted for a small proportion of the above studies. In fact, our study could not determine the efficacy of the combination either because the 30-day mortality in our study was 29%, similar to the findings of Tumbarello, M et al. Colistin susceptibility testing was not performed because of retrospective studies and testing costs, which may have contributed to colistin insensitivity in 31 patients, and the combination therapy may have switched to CAZ/AVI monotherapy. Second, compared with other studies, most of the patients in our study were critically ill (the mean Apache II and SOFA score were 22 and 10, respectively), and CAZ/AVI combined with colistin may not be effective as an end-stage salvage therapy. Therefore, our study cannot confirm the efficacy of combination therapy, but it may be more effective in critically ill patients.

In terms of the type of infection, the most common type of infection in our study was lung infection, accounting for 87.1%, while urinary tract infection (UTI) and blood infection (BSI) accounted for only 12.9%, which was also consistent with the expected results observed in our clinical practice and the results reported in the relevant literature. Viderman, D's [37] observational study of ICU-associated infections in Kazakhstan showed that the incidence of ventilator-associated pneumonia (VAP) was greater than UTI and BSI. Therefore, the types of infections we studied were mainly respiratory infections, which were primarily related to the population we included. All the patients were seriously ill and admitted to the ICU, and the vast majority were mechanically ventilated (90.3%), which dramatically increased the likelihood of pulmonary infection. The mortality rate of VAP in previous studies ranged from 20 to 60% [38, 39], and the results of our study were also within this range. However, because the patients were critically ill and the 30-day mortality rate was only 29%, the overall therapeutic effect of this combination in treating respiratory tract infection is acceptable, but whether it can reduce mortality still needs more research support. In addition, the two patients with bloodstream infection in our study died, which may be related to multiple organ failure caused by sepsis. However, because of the small number of cases of this type, the effect of combination therapy is inaccurate.

The use of colistin has been increasing worldwide in recent years, and the major limiting factors is nephrotoxicity, which is dose-dependent and reversible. Permanent renal damage is rarely seen [40], with rates ranging from 20 to 76% [41]. Nephrotoxicity is associated with age, gender, hypoalbuminemia, hyperbilirubinemia, nephrotoxic drug use, various comorbidities, and high-dose and long-term use of colistin [41]. The ROC curve in our study was used to analyze the correlation between the days of combined treatment and AKI. The cut-off value was 8.5 days, and the area under the curve was 0.717, which had a high prediction accuracy for nephrotoxicity, but the small sample size would reduce the accuracy of the test. In addition, AVI is metabolized by the kidney, which is related to AKI to a certain extent. Although Shields, R. K et al. ‘s [42] previous study suggested that the nephrotoxicity of CAZ/AVI is less than colistin, the nephrotoxicity of CAZ/AVI still needs to be considered. Actually, thirteen patients had renal dysfunction and were already on CRRT before using this combination, and we had no direct evidence of an increase in AKI. Diarrhea was also observed in two patients, but cultures for clostridium difficile were negative, possibly related to the use of CAZ/AVI [43]. Anaphylaxis [43] is another side effect reported in the phase 3 trial of cifortal but was not observed in our study. Neurotoxicity [44] of colistin and CAZ/AVI was also a side effect, but neurotoxicity was not observed in our study or could not be assessed due to psychiatric factors in the patients.

Currently, CR-GNB is widely spread worldwide and poses a severe threat to public health [45], which is a great challenge to clinicians and pharmacists, and the choice of antibiotics has become the key to solving the problem. At present, the study of CAZ/AVI combined with colistin in the treatment of CR-GNB infection mainly focuses on in vitro experiments, and it is found that the two have synergistic effects. Our study has made a preliminary exploration of colistin combined with CAZ/AVI in the treatment of CR-GNB infection, but we did not study the timing of combination therapy, comparison of monotherapy, treatment of infection with other pathogenic pathogens, and accurate evaluation of drug dose. In the future, we will conduct prospective studies to study its efficacy further. More relevant reports will support our study results in the future and provide new options for treating CR-GNB infection in clinical practice.

There are still limitations to the current use of this combination in clinical practice. First, clinicians do not prioritize this combination because of its high cost. Second, combination therapy is mainly used to treat super bacterial infections, and most CR-GNB is still clinically sensitive to colistin, tigecycline, and CAZ/AVI. Clinicians should consider the combination only when the above treatments are not effective. Third, the existing studies on the combination therapy of this group are few, and its efficacy and side effects are not exact, which limits its wide application. Fourth, in many cases, this combination is used as salvage therapy in the ICU. However, whether it accelerates the progression of bacterial resistance in patients with end-stage disease is unknown.

Our study had several limitations. First, because of its retrospective, single-center observational design, indication bias must be considered, and the small sample size prevented the taking of patient co-morbidities into consideration with logistic regression analysis and may have affected the study results. Second, most patients were only given colistin combined with CAZ/AVI for anti-infection, but some patients were given antifungal or anti-Gram-positive coccal drugs, which may have influenced our research results. Third, due to the limitations of the retrospective study, we did not have colistin susceptibility testing. Some patients may have false susceptibility, and the effect of combination therapy only shows the effect of CAZ/AVI monotherapy, which is a critical defect of our study. In the future prospective study, we will pay more attention to colistin susceptibility testing and use colistin in combination under the condition of sensitivity.

## Conclusion

The combination of CAZ/AVI and colistin has potential efficacy in patients with CR-GNB infection, but more studies are needed to determine whether it can reduce 30-day mortality rates and increase 7-day microbial clearance. At the same time, the adverse reactions of combination therapy should not be ignored.

## Data Availability

All data generated or analyzed during this study are included in this published article. The datasets used and/or analysed during the current study are available from the corresponding author on reasonable request.

## Abbreviations

CAZ/AVI: Ceftazidime/avibactam
CR-GNB: Carbapenem-resistant Gram-negative bacilli
CRP: C-reactive protein
GNB: Gram-negative bacteria
MICs: Minimum inhibitory concentrations
PCT: Procalcitonin
WBC: White blood cell
CRRT: Continuous renal replacement therapy
UTI: Urinary tract infection
BSI: Bloodstream infection
VAP: Ventilator-associated pneumonia
ROC: Receiver operating characteristic curve
AUC: Area under the curve
Apache II score: Acute Physiology and Chronic Health Evaluation
SOFA: Sequential Organ Failure Assessment
